# Metabolic Syndrome and Physical Inactivity May Be Shared Etiological Agents of Prostate Cancer and Coronary Heart Diseases

**DOI:** 10.3390/cancers14040936

**Published:** 2022-02-14

**Authors:** Antonio Cicione, Aldo Brassetti, Riccardo Lombardo, Antonio Franco, Beatrice Turchi, Simone D’Annunzio, Antonio Nacchia, Andrea Tubaro, Giuseppe Simone, Cosimo De Nunzio

**Affiliations:** 1 Department of Urology, Sant’Andrea Hospital, Via di Grottarossa 1035, 00189 Rome, Italy; acicione@libero.it (A.C.); rlombardo@me.com (R.L.); anto.franco@hotmail.it (A.F.); be.turchi@gmail.com (B.T.); simone.dannunzio@uniroma1.it (S.D.); antonionacchia7@gmail.com (A.N.); andrea.tubaro@mac.com (A.T.); cosimodenunzio@virgilio.it (C.D.N.); 2 Department of Urology, IRCCS “Regina Elena” National Cancer Institute, Via Elio Chianesi 53, 00144 Rome, Italy; puldet@gmail.com

**Keywords:** prostate cancer, major cardiac events, physical activity, cardiovascular diseases

## Abstract

**Simple Summary:**

As metabolic syndrome (MetS) and a sedentary lifestyle are associated with an increased risk of prostate cancer (PCa) and cardiovascular diseases (CVDs), the 2 conditions may share common causes. We investigated the association between CVDs and PCa. Clinical data from patients undergone prostate biopsy were collected, physical activity (PA) was assessed and coronary heart diseases (CHDs) recorded. PCa was diagnosed in 395/955 men and 238 were aggressive tumors. Although CHDs were more common among PCa-patients (9.4% vs. 7.5%) the difference was not statistically significant and no difference was observed between low- and high-grade subgroups (9.5% vs. 9.2%). PA significantly reduced the risk of PCa diagnosis and aggressiveness while MetS only increased the risk of being diagnosed with cancer. CHDs were associated neither with tumor diagnosis nor aggressiveness. MetS and PA are strong predictors of PCa. We failed to prove a significant association between PCa and CHDs.

**Abstract:**

As metabolic syndrome (MetS) and a sedentary lifestyle have been associated with an increased risk of developing both prostate cancer (PCa) and cardiovascular diseases (CVDs), the 2 conditions may share a common etiology. We aimed at investigating the association between CVDs and PCa. A retrospective analysis was performed. Our dataset on patients undergone systematic prostate biopsy was searched for histopathologic and clinical data. The physical activity (PA) scale for the elderly (PASE) was collected. Coronary heart diseases (CHDs) were recorded. Prognostic Grade Group ≥3 tumors were defined as high-grade (HG). The association between MetS, PA, CHDs and PCa was assessed using logistic regression analyses. Data on 955 patients were collected; 209 (22%) presented with MetS, 79 (8%) with CHDs. PCa was diagnosed in 395 (41.3%) men and 60% (*n* = 238) presented with an high-grade tumor. CHDs were more common among PCa-patients (9.4% vs. 7.5%; *p* = 0.302) but the difference was not statistically significant. No difference was observed between low- and high-grade subgroups (9.5% vs. 9.2%; *p* = 0.874). PASE independently predicted PCa diagnosis (OR: 0.287; *p* = 0.001) and HG-PCa (OR: 0.165; *p* = 0.001). MetS was an independent predictor of HG-PCa only (OR: 1.50; 95% CI: 1.100–2.560; *p* = 0.023). CHDs were not associated with tumor diagnosis and aggressiveness.

## 1. Introduction

Cardiovascular (CV) diseases (CVDs) represent a major cause of morbidity and mortality worldwide, with a lifetime risk exceeding 60%. More than 2200 Americans die of CVDs daily, one every 40 s [1]. Epidemiological projections are not reassuring as it has been estimated that CVDs incidence will escalate by 10% between 2010 and 2030 due to increasing rates of obesity, hypertension and diabetes [2]. These diseases of affluence, which are strongly related to a sedentary lifestyle, are key-components of metabolic syndrome (MetS) [3], a pro-inflammatory systemic condition which increases by two folds the risk of CVDs and related mortality [4].

Interestingly, *Syndrome X* and physical inactivity are also associated with prostatic diseases [5]. Men with metabolic issues, in fact, are thought to have alterations in vascular supply and innervation of several tissues, including bladder and prostate, which may cause lower urinary tract symptoms (LUTS) [3]. Similarly, *metaflammation* that characterizes sedentary MetS-patients may generate a pro-proliferative micro-tissue environment potentially leading to prostate cancer (PCa) [6], which in turn is the second leading cause of cancer death in men in the United States [7] and whose prevalence is significantly higher in this specific population [8].

In light of this, considering that a recent systematic review affirmed that patients with LUTS are at increased risk of coronary heart diseases (CHDs) [3] we hypothesized that these CV events could also be associated with PCa, and metabolic disorders may play a role in a shared etiologic mechanism. The present study aimed at investigating the possible association between CHDs and PCa diagnosis and aggressiveness and the role of MetS and physical activity (PA) in this pathogenetic pathway.

## 2. Materials and Methods

### 2.1. Patients and Dataset

After institutional review board approval, a retrospective analysis of our prospectively maintained database was performed. Data of 1332 patients undergone systematic prostate biopsy from January 2012 to September 2017 for elevated prostate specific antigen (PSA) values (≥4 ng/mL) and/or a suspected digital rectal examination (DRE) were selected for the analysis. Men that received more than one biopsy within the study timeframe and those with a history of previous prostate surgery were excluded. 

Age and anthropometric parameters were assessed according to standardized methods and recorded from all patients. Waist circumference was measured, using a standard measurement strip with the patients standing and breathing normally, at the midway between the lowest rib margin and iliac crest [9]. BMI was calculated as weight in kilograms divided by height in meters, squared (kg/m^2^). Obesity was defined as BMI ≥ 30 kg/m^2^. Resting blood pressure was recorded as the first and fifth Korotkof sounds by auscultation methods [10]. Fasting (8 h) blood samples were drawn from all patients during the preoperative assessment evaluation and analyzed for blood glucose, HDL cholesterol, triglycerides, total Prostate Specific Antigen (PSA) [11]. The average daily energy expenditure was estimated for each patient, and these data were also included in the purpose-built dataset. The assessment was performed at the time of prostate biopsy, through the administration of the Physical Activity Scale for the Elderly (PASE), a questionnaire that measures frequency and duration of self-reported physical activity in the adults, and is comprised of items regarding occupational, household and leisure activities [8,12].

The presence of metabolic syndrome (MetS) was defined according to Adult Treatment Panel III (ATPIII) criteria [13]. Within 15 days before prostate biopsy, all patients were requested to undergo a resting electrocardiogram to rule out hidden heart diseases. These data, together with a personal history of Q-waves, myocardial infarctions or coronary disease requiring heart revascularizations (by means of bypass graft surgery or percutaneous transluminal angioplasty) were used to diagnose or confirm the presence of CHDs [3,14]. International Society of Urological Pathology (ISUP) Prognostic Grade Group (PGG) ≥3 tumors were defined as high-grade prostate cancers (HG-PCa) [15].

### 2.2. Statistical Analysis

Frequencies and proportions were used to report categorical variables that were compared by means of the Chi-squared test. Continuous variables were presented as median and interquartile ranges (IQRs) and were compared using either the Mann Whitney U test or Kruskal Wallis one-way based on their normal or not-normal distribution, respectively (normality of the distribution of variables was tested by the Kolmogorov Smirnov test). Predictors of PCa diagnosis and high grade disease were identified by means of univariable and multivariable logistic regression models (inclusion method): odds ratios and 95% confidence intervals were reported. An alpha value of 5% was considered as threshold for significance. Statistical analysis was performed using Statistical Package for Social Science 24.0 software (SPSS Inc., Chicago, IL, USA).

## 3. Results

Overall, 377 men were excluded from the analysis: 220/1332 (16%) because of multiple biopsies, 75/1332 (6%) for a personal history of prostate surgery and 82 (6%) for missing variables. Consequently, we included data on 955 consecutive patients, with a median age of 65 (IQR: 60–75) years and BMI of 26 (IQR: 21.4–30.2) kg/m^2^ (Table 1). Diabetes and hypertension were observed in 89 (9.4%) and 500 (52.3%) men, respectively; 209 (21.8%) were diagnosed with MetS. Overall, 79 (8.2%) patients reported a CHD in their medical history: 16 Q-waves, 30 myocardial infarction, 33 heart revascularization (Table 1). PCa was diagnosed in 395 (41.3%) men and 60% of these (*n* = 238) presented with an high-grade tumor. No statistically significant difference in terms of CHDs rate was observed between patients with or without PCa (9.4% vs. 7.5%; *p* = 0.302) (Table 1); the observed rate was comparable also in low- and high-grade subgroups (9.5% vs. 9.2%; *p* = 0.874). 

At multivariable analysis, age (OR: 1.03; 95% CI: 1.012–1.055; *p* = 0.012), prostate volume (PV) (OR: 0.97; 95% CI: 0.966–0.980; *p* = 0.001), PSA at diagnosis (OR: 1.10; 95% CI: 1.063–1.142; *p* = 0.001) and PASE score (OR: 0.28; 95% CI: 0.124–0.668; *p* = 0.001) were independent predictors of PCa diagnosis (Table 2). These variables independently predicted HG-PCa too, together with MetS (OR: 1.50; 95% CI: 1.100–2.560; *p* = 0.023) (Table 3). A personal history of CHDs was associated neither with PCa diagnosis nor with high grade disease (Table 2 and Table 3).

## 4. Discussion

CVDs and PCa are two of the most urgent health challenges of this century, in Western Countries, and their prevalence is bound to increase [2] because of the so-called Metabolic syndrome pandemic [16], which in turn is a consequence of population ageing and sedentary lifestyle [17]. 

The systemic inflammatory status that characterizes MetS-patients may be a shared etiological agent of these diseases [4,18,19] and a recent meta-analysis showed an association between inflammatory markers and the risk of PCa [20]. Most authors advocate that chronic prostatic inflammation may induce carcinogenesis through several mechanisms including direct cellular and genomic damage, local immunosuppression and creation a pro-proliferative micro-tissue environment [21,22]. In a recent review, Silveira Rossi et al. reported that diabetes mellitus, hypertension and obesity are responsible for a chronic low-grade systemic inflammatory state which in turn is directly related to atherosclerosis and CVDs incidence [6]. This *metaflammation* causes the surge of reactive oxygen species which result in post-translational alterations of proteins, lipids and DNA [6]. Chronically inflamed adipocytes play a key role in this pathologic process by secreting pro-inflammatory cytokines, such as interleukin-1β, -6, and tumor necrosis factor-α which are relevant for the pathogenesis of certain neoplasms (colorectal cancers among others) [23]. 

Our previous findings and a recent meta-analysis (which included 24 studies and 132,589 participants) concluded that MetS is associated with an increased risk of HG cancers at prostate biopsy, adverse features at final pathology, disease recurrence and cancer-specific mortality [11,18,24,25,26]. Results from the present study confirmed that MetS only increased the risk of HG-PCa (OR: 1.50; 95%CI: 1.100–2.560; *p* = 0.023) (Table 2 and Table 3). One third of men with elevated PSA levels present at least with one of MetS components [27]. Individuals with hypertension show a higher risk of being diagnosed with PCa [28] and the use of calcium-channel-blockers has been associated with an increased incidence of this tumor [29]: it is hypothesized that these drugs promote carcinogenesis by affecting the normal function of Cav3.1 channels which contribute to tumor repression and apoptosis promotion [29]. Advanced glycosylation end products, whose levels are particularly high in diabetic patients, are known to be responsible for cellular dysfunction [6]. Unexpectedly, however, diabetes seems inversely associated with the risk of PCa [30]. Though, men with type 2 diabetes typically present with low PSA levels, which might result in cancer underdiagnosis [31] as supported by the REDUCE trial (where participants were required to undergo biopsy regardless of PSA values) which failed to show an association between diabetes and PCa risk [32]. Conflicting results were reported concerning the impact of obesity on PCa risk. Giovannucci et al. found BMI positively associated with high-risk tumors and postulated that this could be due to the compromised balance between serum concentration of estrogen, testosterone, insulin and insulin-like growth factor-1, which is affected by adipose tissue [33]. Similarly, a meta-analysis of 17 cohort studies, demonstrated that obesity was associated with an increased risk of aggressive PCa and cancer-specific mortality [34]. Conversely, recent reviews failed to detect an association between visceral obesity and PCa development [35]. In particular, few case-control studies highlighted that a higher BMI is associated with a reduced risk of PSA-detected PCa [36,37]. Again, these observations might in part be explained by underdiagnosis as a 5 kg/m^2^ increase in BMI was associated with a 6% reduction in PSA levels [38], as per *obesity-related plasma hemodiluition* [39].

While the protective role of PA on the risk of CVDs is already supported by grounded evidences, its possible association with PCa prevention has been assessed only recently, and conflicting results were reported [40,41]. It has been postulated that exercise is capable of increasing telomere length and modulating gene expression responsible for protein intracellular transportation, metabolism and phosphorylation [42]. PA could also improve insulin-resistance and interfere with the levels of various circulating tumor-promoting proteins such as insulin-like growth factor-1 [43]. Finally, intensive training reduces adiposity, thus decreasing levels of blood levels of proinflammatory adipokines [44]. We already showed that an active lifestyle reduces the risk of cancer diagnosis and high-grade tumor at biopsy [12] and even reclassification during active surveillance [45]. Other authors showed that increasing levels are associated with a reduced risk for tumor recurrence and disease-specific death after primary treatment [46]. A recent systematic review by the European Association of Urology Section of Oncological Urology even concluded that regular physical activity reduces the risk of local and systemic disease progression, cancer-specific and overall mortality [47]. For these reasons, exercise has been included in the ASCO (American Society of Clinical Oncology) Clinical Practice Guidelines on PCa. Results from the present study confirmed our previous findings as PA significantly reduced the risk of PCa diagnosis and aggressiveness (Table 2 and Table 3).

Being both highly prevalent in Western Countries and considering their association with a sedentary lifestyle and the diseases of affluence, CVDs and PCa may have a common pathophysiological pathway, but this hypothesis has been scarcely investigated. According to a recent post-hoc analysis from the RADICAL PC study, two thirds of men with PCa were at high CV risk [48]. Correspondingly, another post-hoc analysis from the REDUCE trial highlighted that patients with a medical history of CHD show a 35% increased risk of being diagnosed with PCa (OR = 1.35, 95% CI: 1.08–1.67, *p* = 0.007) [49]. Similarly, we previously reported that patients with a moderate/high CV risk present an increased risk of HG-PCa (OR: 2.154, 95% CI: 1.076–4.314; *p* = 0.030) [50]. Results from the present study are in contrast with these observations. Actually, in the present study we chose to assess the association between PCa and CHDs. In fact, Q-waves, myocardial infarction and coronary disease requiring heart revascularization are three different clinical manifestations of the same pathophysiologic process that leads to CVDs [3]. Only 8.2% of our sample had a history of CHD while higher rates were reported in other series investigating the association between CHD and LUTS [18]. Although these cardiovascular events were more common among PCa-patients compared to controls (9.4% vs. 7.5%; *p* = 0.302) the difference did not reach statistical significance; the same occurred in high-grade and low-grade subgroups (9.5% vs. 9.2%; *p* = 0.874). At multivariate analysis, therefore, CHDs were not independent predictors of PCa diagnosis and high-grade tumor at biopsy.

Recently, the possible association between CHDs and PCa-medications has been extensively investigated, based on the assumption that men who have undergone bilateral orchiectomy are at increased risk of CVDs [51]. However, the influence of gonadotropin-releasing-hormone (GnRH) analogs on CV toxicity remain controversial. Several studies provided evidence that their use significantly increases the risk of myocardial infarction and stroke [52,53] while results from randomized clinical trials reported no differences [54,55]. Also a recent meta-analysis found no added risk of CV mortality in patients taking GnRH agonist vs. controls (RR = 0.93, *p* = 0.041) [56].

This study suffers from limitations inherent to its retrospective design. Regarding this, several detailed sociodemographic data which could affect the risk of CHDs (such as marital status, education and work experience) were not recorded on patients files and could not be extracted. Also the smoking status was not available in most of medical charts, thus it was not considered among the possible variables affecting the risk of PCa and CVDs. Another possible limitation of the present study is the use of the Physical Activity Scale for the Elderly to measure the average daily energy expenditure: this tool, in fact, was initially conceived for patients older that 65 years while a quarter of our study population is younger that 60 years. This questionnaire, however, is not only indicated for retired men as it investigate occupational, household and leisure activities. In fact, it was already successfully administered to cohorts of patients of the same age [8,12,45,57] or even younger [58,59]. Moreover, although a pro-inflammatory systemic condition is thought to be the shared etiological agent of CVDs and PCa, extent and degree of prostatic inflammation were not routinely assessed at the time of prostate biopsy. Study design with a convenience sample makes causal inferences difficult; borderline *p*-values should be interpreted with caution, with careful attention to both internal consistency and biological plausibility; and that residual confounding due to unknown or incompletely measured factors cannot be excluded.

To the best of our knowledge, ours is the first study investigating the association between CHDs and PCa incidence and aggressiveness. With this regard, one strength of our research is that possible hidden heart diseases were ruled out requiring every patient to undergo an electrocardiogram just before prostate biopsy, so that the identification of patients with heart disease was not only based on self-reported medical history, as previously done by other authors [14].

## 5. Conclusions

We confirmed that patients with MetS have an increased risk of being diagnosed with HG-PCa, compared to controls. Also the protective role of PA was affirmed, as higher PASE scores were associated with a reduced risk of cancer diagnosis and aggressiveness. Although CHDs are significantly more common among PCa-patients, regardless tumor grade, we failed to prove the predictive value of these cardiovascular events on cancer diagnosis.

## Figures and Tables

**Table 1:** Patients’ characteristics and outcomes, according to prostate cancer diagnosis.

Patients’ Characteristics and Outcomes	Overall	No PCa	PCa	*p*
	*n* = 955	*n* = 560 (59%)	*n* = 395 (41%)	-
Age, years	65 (60–75)	66 (59–71)	70 (66–74)	0.002
BMI, kg/m^2^	26 (24.1–30.2)	26.3 (24.5–29.2)	26.2 (24.2–29.5)	0.572
MetS, *n* (%)	209 (22%)	112 (20%)	97 (24%)	0.123
Hypertension, *n* (%)	220 (23%)	130 (23%)	90 (23%)	0.876
Tryglicerides, mg/dL	125 (88–172)	120 (80–156)	128 (97–158)	0.345
HDL, mg/dL	50 (40–57)	51 (39–56)	48 (35–56)	0.635
Waist, cm	101 (95–105)	100 (96–110)	102 (95–115)	0.898
Glucose level, g/dL	95 (80–107)	90 (84–115)	98 (85–112)	0.786
Prostate Volume, mL	50 (36–69)	58 (45–90)	40 (31–60)	0.001
PSA at baseline, ng/mL	6 (3.2–12)	5.6 (4.4–8.3)	6.8 (5.1–10)	0.001
PASE score	120.5 (80–170)	125 (83–190)	108 (70–145)	0.001
Personal history of CHD, *n* (%)	79 (8.2%)	42 (7.5%)	37 (9.4%)	0.302
Q-waves, *n* (%)	16 (2%)	9 (2%)	7 (2%)	0.792
Myocardial infarction, *n* (%)	30 (3%)	17 (3%)	13 (3%)	0.482
Revascularization, *n* (%)	33 (3%)	20 (4%)	13 (3%)	0.452
HG-PCa, *n* (%)	238 (25%)	-	238 (60%)	-

Data are presented as Median (IQR) BMI = body mass index, MetS = metabolic syndrome, PASE = Physical Activity Scale for the Elderly, CHD = coronary heart disease, HG-PCa = high grade prostate cancer.

**Table 2:** Univariable and multivariable logistic regression analyses to identify predictors of prostate cancer diagnosis.

Predictors of Prostate Cancer Diagnosis	Univariable Analysis				Multivariable Analysis			
	OR	95% CI		*p* Value	OR	95% CI		*p*-Value
		Lower	Higher			Lower	Higher	
Age, years	1.05	1.021	1.083	0.002	1.03	1.012	1.055	0.012
BMI, kg/m^2^	1.03	0.973	1.114	0.540	-	-	-	-
Metabolic Syndrome	1.16	0.772	1.802	0.454	-	-	-	-
Prostate volume, mL	0.96	0.951	0.972	0.001	0.97	0.966	0.980	0.001
PSA at baseline, ng/mL	1.07	1.042	1.093	0.001	1.10	1.063	1.142	0.001
PASE score	0.33	0.100	0.782	0.001	0.28	0.124	0.668	0.001
Personal hystory of CHD	1.08	0.632	1.904	0.828	-	-	-	-

BMI = body mass index, MetS = metabolic syndrome, PASE = Physical Activity Scale for the Elderly, CHD = coronary heart disease, HG-PCa = high grade prostate cancer.

**Table 3:** Unilabiate and multivariate logistic regression analyses to identify predictors of high grade prostate cancer.

Predictors of High Grade Prostate Cancer	Univariable Analysis				Multivariable Analysis			
	OR	95% CI		*p* Value	OR	95% CI		*p*-Value
		Lower	Higher			Lower	Higher	
Age, years	1.04	1.001	1.060	0.032	1.05	1.017	1.083	0.003
BMI, kg/m^2^	1.10	0.981	1.232	0.234	-	-	-	-
Metabolic Syndrome	2.02	1.301	3.303	0.001	1.50	1.100	2.560	0.023
Prostate volume, mL	0.96	0.951	0.993	0.001	0.98	0.978	0.999	0.008
PSA at baseline, ng/mL	1.08	1.062	1.112	0.001	1.09	1.038	1.160	0.001
PASE score	0.23	0.090	0.435	0.001	0.16	0.024	0.576	0.001
Personal hystory of CHD	0.83	0.773	3.302	0.454	-	-	-	-

BMI = body mass index, MetS = metabolic syndrome, PASE = Physical Activity Scale for the Elderly, CHD = coronary heart disease, HG-PCa = high grade prostate cancer.

## Data Availability

Data supporting reported results are deposited at https://gbox.garr.it/ (accessed on 4 February 2022).

## References

[1] Zaman S., Goldberger J.J., Kovoor P. (2019). Sudden Death Risk-Stratification in 2018–2019: The Old and the New. Heart Lung Circ..

[2] Mozaffarian D. (2016). Dietary and Policy Priorities for Cardiovascular Disease, Diabetes, and Obesity: A Comprehensive Review. Circulation.

[3] Gacci M., Corona G., Sebastianelli A., Serni S., De Nunzio C., Maggi M., Vignozzi L., Novara G., McVary K.T., Kaplan S.A. (2016). Male Lower Urinary Tract Symptoms and Cardiovascular Events: A Systematic Review and Meta-analysis. Eur. Urol..

[4] Mottillo S., Filion K.B., Genest J., Joseph L., Pilote L., Poirier P., Rinfret S., Schiffrin E.L., Eisenberg M.J. (2010). The metabolic syndrome and cardiovascular risk: A systematic review and meta-analysis. J. Am. Coll. Cardiol..

[5] De Nunzio C., Kramer G., Marberger M., Montironi R., Nelson W., Schröder F., Sciarra A., Tubaro A. (2011). The controversial relationship between benign prostatic hyperplasia and prostate cancer: The role of inflammation. Eur. Urol..

[6] Silveira Rossi J.L., Barbalho S.M., Reverete de Araujo R., Bechara M.D., Sloan K.P., Sloan L.A. (2021). Metabolic syndrome and cardiovascular diseases: Going beyond traditional risk factors. Diabetes Metab. Res. Rev..

[7] Rawla P. (2019). Epidemiology of Prostate Cancer. World J. Oncol..

[8] De Nunzio C., Nacchia A., Cicione A., Cindolo L., Gacci M., Cancrini F., Castellan P., Lombardo R., D’Annunzio S., Sarchi L. (2019). Physical Activity as a Protective Factor for Lower Urinary Tract Symptoms in Male Patients: A Prospective Cohort Analysis. Urology.

[9] Abdeen Z., Jildeh C., Dkeideek S., Qasrawi R., Ghannam I., Al Sabbah H. (2012). Overweight and Obesity among Palestinian Adults: Analyses of the Anthropometric Data from the First National Health and Nutrition Survey (1999–2000). J. Obes..

[10] Frese E.M., Fick A., Sadowsky S.H. (2011). Blood Pressure Measurement Guidelines for Physical Therapists. Cardiopulm. Phys. Ther. J..

[11] De Nunzio C., Freedland S.J., Miano R., Trucchi A., Cantiani A., Carluccini A., Tubaro A. (2011). Metabolic syndrome is associated with high grade gleason score when prostate cancer is diagnosed on biopsy. Prostate.

[12] De Nunzio C., Presicce F., Lombardo R., Cancrini F., Petta S., Trucchi A., Gacci M., Cindolo L., Tubaro A. (2016). Physical activity as a risk factor for prostate cancer diagnosis: A prospective biopsy cohort analysis. BJU Int..

[13] Von der Maase H. (2002). Third Report of the National Cholesterol Education Program (NCEP) Expert Panel on Detection, Evaluation, and Treatment of High Blood Cholesterol in Adults (Adult Treatment Panel III) final report. Circulation.

[14] Weisman K.M., Larijani G.E., Goldstein M.R., Goldberg M.E. (2000). Relationship between Benign Prostatic Hyperplasia and History of Coronary Artery Disease in Elderly Men. Pharmacother. J. Hum. Pharmacol. Drug Ther..

[15] De Nunzio C., Pastore A.L., Lombardo R., Simone G., Leonardo C., Mastroianni R., Collura D., Muto G., Gallucci M., Carbone A. (2018). The new Epstein gleason score classification significantly reduces upgrading in prostate cancer patients. Eur. J. Surg. Oncol..

[16] Perletti G., Monti E., Magri V., Cai T., Cleves A., Trinchieri A., Montanari E. (2017). The association between prostatitis and prostate cancer. Systematic review and meta-analysis. Arch. Ital. Urol. Androl..

[17] Myers J., Kokkinos P., Nyelin E. (2019). Physical Activity, Cardiorespiratory Fitness, and the Metabolic Syndrome. Nutrients.

[18] Gacci M., De Nunzio C., Sebastianelli A., Salvi M., Vignozzi L., Tubaro A., Morgia G., Serni S. (2017). Meta-Analysis of metabolic syndrome and prostate cancer. Prostate Cancer Prostatic Dis..

[19] Aoun F., Albisinni S., Chemaly A.K., Zanaty M., Roumeguère T. (2016). In search for a common pathway for health issues in men-the sign of a holmesian deduction. Asian Pac. J. Cancer Prev..

[20] Michels N., van Aart C., Morisse J., Mullee A., Huybrechts I. (2021). Chronic inflammation towards cancer incidence: A systematic review and meta-analysis of epidemiological studies. Crit. Rev. Oncol. Hematol..

[21] Nelson W.G., De Marzo A.M., Isaacs W.B. (2003). Prostate Cancer. N. Engl. J. Med..

[22] De Bono J.S., Guo C., Gurel B., De Marzo A.M., Sfanos K.S., Mani R.S., Gil J., Drake C.G., Alimonti A. (2020). Prostate carcinogenesis: Inflammatory storms. Nat. Rev. Cancer.

[23] Riondino S., Roselli M., Palmirotta R., Della-Morte D., Ferroni P., Guadagni F. (2014). Obesity and colorectal cancer: Role of adipokines in tumor initiation and progression. World J. Gastroenterol..

[24] Morlacco A., Moro F.D., Rangel L.J., Carlson R.E., Schulte P.J., Jeffrey K.R. (2018). Impact of metabolic syndrome on oncologic outcomes at radical prostatectomy. Urol. Oncol. Semin. Orig. Investig..

[25] De Nunzio C., Simone G., Brassetti A., Mastroianni R., Collura D., Muto G., Gallucci M., Tubaro A. (2016). Metabolic syndrome is associated with advanced prostate cancer in patients treated with radical retropubic prostatectomy: Results from a multicentre prospective study. BMC Cancer.

[26] De Nunzio C., Brassetti A., Simone G., Lombardo R., Mastroianni R., Collura D., Muto G., Gallucci M., Tubaro A. (2018). Metabolic syndrome increases the risk of upgrading and upstaging in patients with prostate cancer on biopsy: A radical prostatectomy multicenter cohort study. Prostate Cancer Prostatic Dis..

[27] Sourbeer K.N., Howard L.E., Andriole G.L., Moreira D.M., Castro-Santamaria R., Freedland S.J., Vidal A.C. (2015). Metabolic syndrome-like components and prostate cancer risk: Results from the Reduction by Dutasteride of Prostate Cancer Events (REDUCE) study. BJU Int..

[28] Liang Z., Xie B., Li J., Wang X., Wang S., Meng S., Ji A., Zhu Y., Xu X., Zheng X. (2016). Hypertension and risk of prostate cancer: A systematic review and meta-analysis. Sci. Rep..

[29] Yang H., Yu Y., Hu X., Wang W., Yang X., Liu H., Ren L., Zhang X., Feng X., Liu L. (2020). Association Between the Overall Risk of Prostate Cancer and Use of Calcium Channel Blockers: A Systematic Review and Meta-analysis. Clin. Ther..

[30] Ling S., Brown K., Miksza J.K., Howells L., Morrison A., Issa E., Yates T., Khunti K., Davies M.J., Zaccardi F. (2020). Association of Type 2 Diabetes With Cancer: A Meta-analysis With Bias Analysis for Unmeasured Confounding in 151 Cohorts Comprising 32 Million People. Diabetes Care.

[31] Fukui M., Tanaka M., Kadono M., Imai S., Hasegawa G., Yoshikawa T., Nakamura N. (2008). Serum Prostate-Specific Antigen Levels in Men With Type 2 Diabetes. Diabetes Care.

[32] Wu C., Moreira D.M., Gerber L., Rittmaster R.S., Andriole G.L., Freedland S.J. (2011). Diabetes and prostate cancer risk in the REDUCE trial. Prostate Cancer Prostatic Dis..

[33] Giovannucci E., Liu Y., Platz E.A., Stampfer M.J., Willett W.C. (2007). Risk factors for prostate cancer incidence and progression in the health professionals follow-up study. Int. J. Cancer.

[34] Zhang X., Zhou G., Sun B., Zhao G., Liu D., Sun J., Liu C., Guo H. (2015). Impact of obesity upon prostate cancer-associated mortality: A meta-analysis of 17 cohort studies. Oncol. Lett..

[35] Silveira E.A., Kliemann N., Noll M., Sarrafzadegan N., de Oliveira C. (2021). Visceral obesity and incident cancer and cardiovascular disease: An integrative review of the epidemiological evidence. Obes. Rev..

[36] Dimitropoulou P., Martin R.M., Turner E.L., Lane J.A., Gilbert R., Davis M., Donovan J.L., Hamdy F.C., Neal D.E. (2011). Association of obesity with prostate cancer: A case-control study within the population-based PSA testing phase of the ProtecT study. Br. J. Cancer.

[37] Boehm K., Sun M., Larcher A., Blanc-Lapierre A., Schiffmann J., Graefen M., Sosa J., Saad F., Parent M.É., Karakiewicz P.I. (2015). Waist circumference, waist-hip ratio, body mass index, and prostate cancer risk: Results from the North-American case-control study Prostate Cancer & Environment Study. Urol. Oncol. Semin. Orig. Investig..

[38] Harrison S., Tilling K., Turner E.L., Martin R.M., Lennon R., Lane J.A., Donovan J.L., Hamdy F.C., Neal D.E., Bosch J.L.H.R. (2020). Systematic review and meta-analysis of the associations between body mass index, prostate cancer, advanced prostate cancer, and prostate-specific antigen. Cancer Causes Control.

[39] Bañez L.L., Hamilton R.J., Partin A.W., Vollmer R.T., Sun L., Rodriguez C., Wang Y., Terris M.K., Aronson W.J., Presti J.C. (2007). Obesity-Related Plasma Hemodilution and PSA Concentration Among Men With Prostate Cancer. JAMA.

[40] Krstev S., Knutsson A. (2019). Occupational Risk Factors for Prostate Cancer: A Meta-analysis. J. Cancer Prev..

[41] Benke I.N., Leitzmann M.F., Behrens G., Schmid D. (2018). Physical activity in relation to risk of prostate cancer: A systematic review and meta-analysis. Ann. Oncol..

[42] Ornish D., Magbanua M.J.M., Weidner G., Weinberg V., Kemp C., Green C., Mattie M.D., Marlin R., Simko J., Shinohara K. (2008). Changes in prostate gene expression in men undergoing an intensive nutrition and lifestyle intervention. Proc. Natl. Acad. Sci. USA.

[43] Ornish D., Weidner G., Fair W.R., Marlin R., Pettengill E.B., Raisin C.J., Dunn-Emke S., Crutchfield L., Jacobs F.N., Barnard R.J. (2005). Intensive lifestyle changes may affect the progression of prostate cancer. J. Urol..

[44] Schenk J.M., Neuhouser M.L., Beatty S.J., VanDoren M., Lin D.W., Porter M., Gore J.L., Gulati R., Plymate S.R., Wright J.L. (2019). Randomized trial evaluating the role of weight loss in overweight and obese men with early stage prostate Cancer on active surveillance: Rationale and design of the Prostate Cancer Active Lifestyle Study (PALS). Contemp. Clin. Trials.

[45] Brassetti A., Ferriero M., Napodano G., Sanseverino R., Badenchini F., Tuderti G., Anceschi U., Bove A., Misuraca L., Mastroianni R. (2021). Physical activity decreases the risk of cancer reclassification in patients on active surveillance: A multicenter retrospective study. Prostate Cancer Prostatic Dis..

[46] Wekesa A., Harrison M., Watson R.W. (2015). Physical activity and its mechanistic effects on prostate cancer. Prostate Cancer Prostatic Dis..

[47] Brookman-May S.D., Campi R., Henríquez J.D.S., Klatte T., Langenhuijsen J.F., Brausi M., Linares-Espinós E., Volpe A., Marszalek M., Akdogan B. (2019). Latest Evidence on the Impact of Smoking, Sports, and Sexual Activity as Modifiable Lifestyle Risk Factors for Prostate Cancer Incidence, Recurrence, and Progression: A Systematic Review of the Literature by the European Association of Urology Section of Oncological Urology (ESOU). Eur. Urol. Focus.

[48] Leong D.P., Fradet V., Shayegan B., Duceppe E., Siemens R., Niazi T., Klotz L., Brown I., Chin J., Lavallee L. (2020). Cardiovascular Risk in Men with Prostate Cancer: Insights from the RADICAL PC Study. J. Urol..

[49] Thomas J.A., Gerber L., Bañez L.L., Moreira D.M., Rittmaster R.S., Andriole G.L., Freedland S.J. (2012). Prostate cancer risk in men with baseline history of coronary artery disease: Results from the reduce study. Cancer Epidemiol. Biomark. Prev..

[50] De Nunzio C., Truscelli G., Trucchi A., Petta S., Tubaro M., Gacci M., Gaudio C., Presicce F., Tubaro A. (2016). Metabolic abnormalities linked to an increased cardiovascular risk are associated with high-grade prostate cancer: A single biopsy cohort analysis. Prostate Cancer Prostatic Dis..

[51] Keating N.L., O’Malley A.J., Freedland S.J., Smith M.R. (2010). Diabetes and Cardiovascular Disease During Androgen Deprivation Therapy: Observational Study of Veterans With Prostate Cancer. J. Natl. Cancer Inst..

[52] Bosco C., Bosnyak Z., Malmberg A., Adolfsson J., Keating N.L., Van Hemelrijck M. (2015). Quantifying Observational Evidence for Risk of Fatal and Nonfatal Cardiovascular Disease Following Androgen Deprivation Therapy for Prostate Cancer: A Meta-analysis. Eur. Urol..

[53] Zhao J., Zhu S., Sun L., Meng F., Zhao L., Zhao Y., Tian H., Li P., Niu Y. (2014). Androgen deprivation therapy for prostate cancer is associated with cardiovascular morbidity and mortality: A meta-analysis of population-based observational studies. PLoS ONE.

[54] Bolla M., Van Tienhoven G., Warde P., Dubois J.B., Mirimanoff R.O., Storme G., Bernier J., Kuten A., Sternberg C., Billiet I. (2010). External irradiation with or without long-term androgen suppression for prostate cancer with high metastatic risk: 10-year results of an EORTC randomised study. Lancet Oncol..

[55] Efstathiou J.A., Bae K., Shipley W.U., Hanks G.E., Pilepich M.V., Sandler H.M., Smith M.R. (2009). Cardiovascular mortality after androgen deprivation therapy for locally advanced prostate cancer: RTOG 85–31. J. Clin. Oncol..

[56] Nguyen P.L., Je Y., Schutz F.A.B., Hoffman K.E., Hu J.C., Parekh A., Beckman J.A., Choueiri T.K. (2011). Association of Androgen Deprivation Therapy With Cardiovascular Death in Patients With Prostate Cancer: A Meta-analysis of Randomized Trials. JAMA.

[57] De Nunzio C., Presicce F., Tubaro A. (2016). Inflammatory mediators in the development and progression of benign prostatic hyperplasia. Nat. Rev. Urol..

[58] Logan S.L., Gottlieb B.H., Maitl S.B., Meegan D., Spriet L.L. (2013). The Physical Activity Scale for the Elderly (PASE) questionnaire; does it predict physical health?. Int. J. Environ. Res. Public Health.

[59] Lindahl M., Hansen L., Pedersen A., Truelsen T., Boysen G. (2008). Self-reported physical activity after ischemic stroke correlates with physical capacity. Adv. Physiother..

